# Supplementary figures and images for: Global importance analysis: An interpretability method to quantify importance of genomic features in deep neural networks
Source: PLoS Comput Biol. 2021 May 13;17(5):e1008925. doi: 10.1371/journal.pcbi.1008925 (PMC8118286; doi:10.1371/journal.pcbi.1008925)

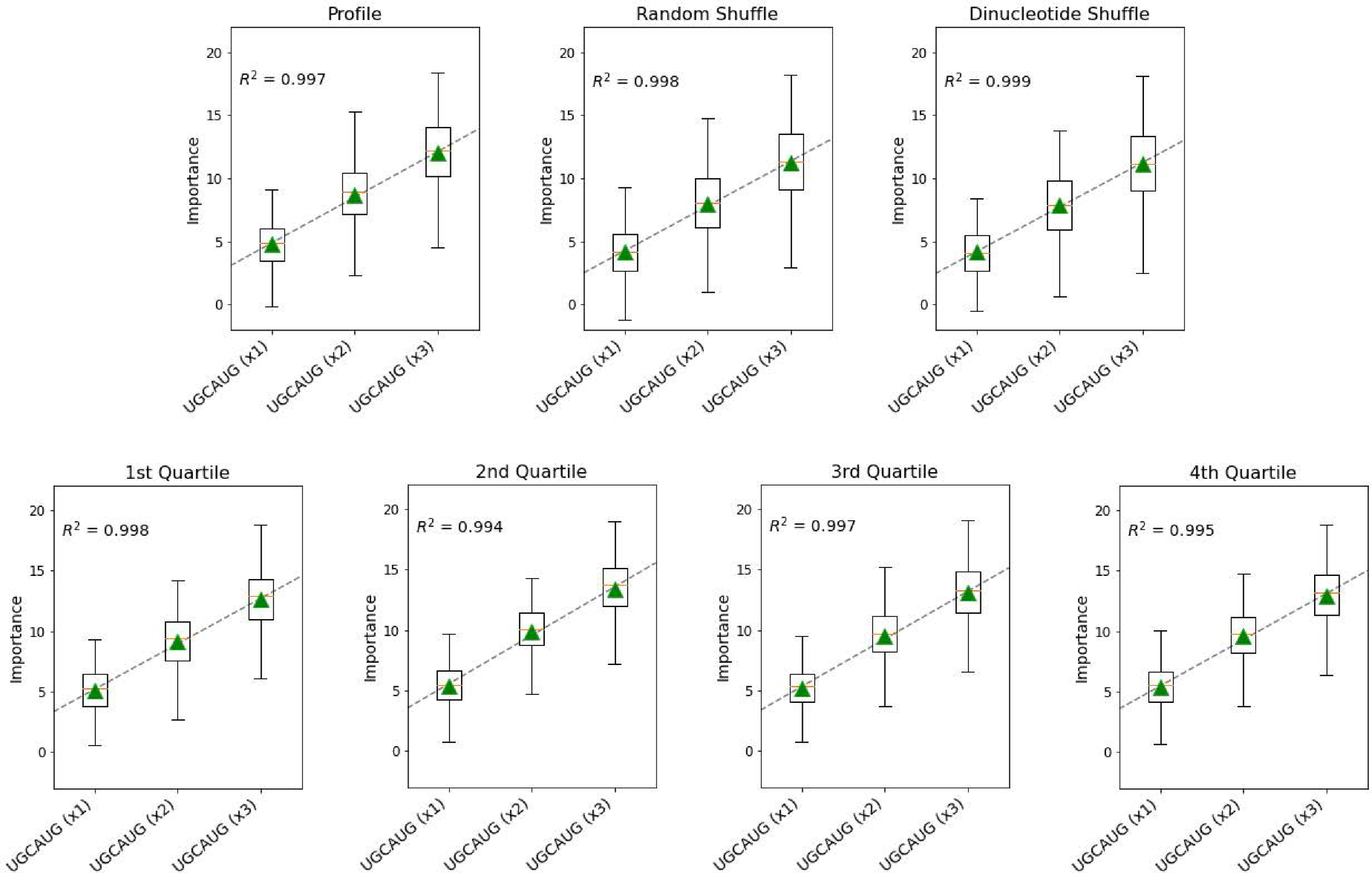

Supplement: S1 Fig — GIA was performed using different models of the approximate data distribution: profile, random shuffle, dinucleotide shuffle, and different binding score quartiles. Box plots of the local importance for synthetic sequences with varying numbers of the canonical RBFOX1 motif (UGCAUG) embedded progressively at positions: 4-9, 11-16, and 18-23. Black dashed line represents a linear fit, red horizontal dashed line represents the median, and green triangles represent the global importance. This demonstrates that GIA is robust across many different models of the approximate data distribution. (TIF) [file pcbi.1008925.s002.tif]

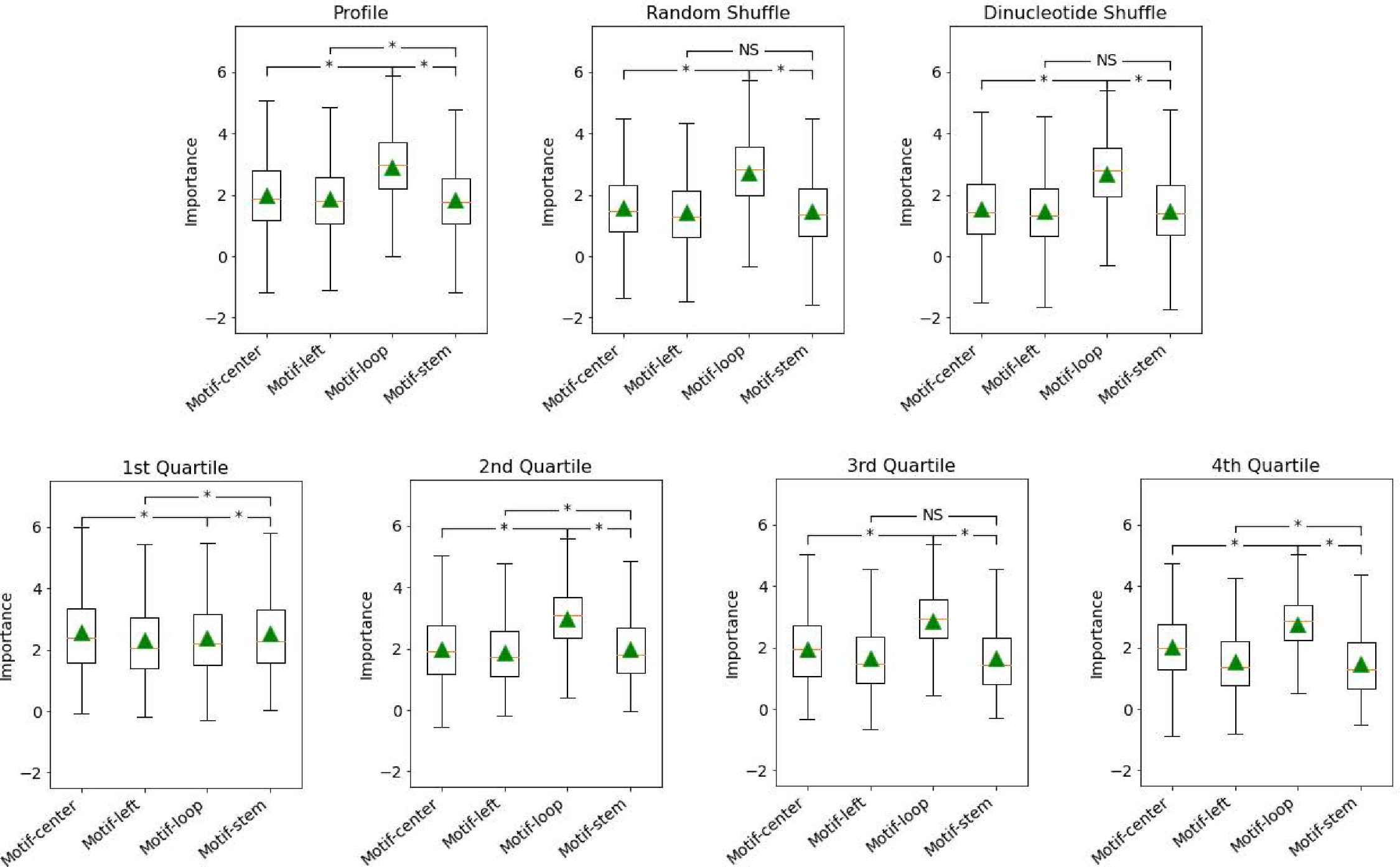

Supplement: S2 Fig — GIA was performed using different models of the approximate data distribution: profile, random shuffle, dinucleotide shuffle, and different binding score quartiles. Box plot of local importance for the top scoring 6-mer pattern, GCUGGC, embedded in the stem and loop region of synthetic sequences designed with a stem-loop structure and in the same positions in random RNA sequences. Green triangles represent the global importance. (TIF) [file pcbi.1008925.s003.tif]

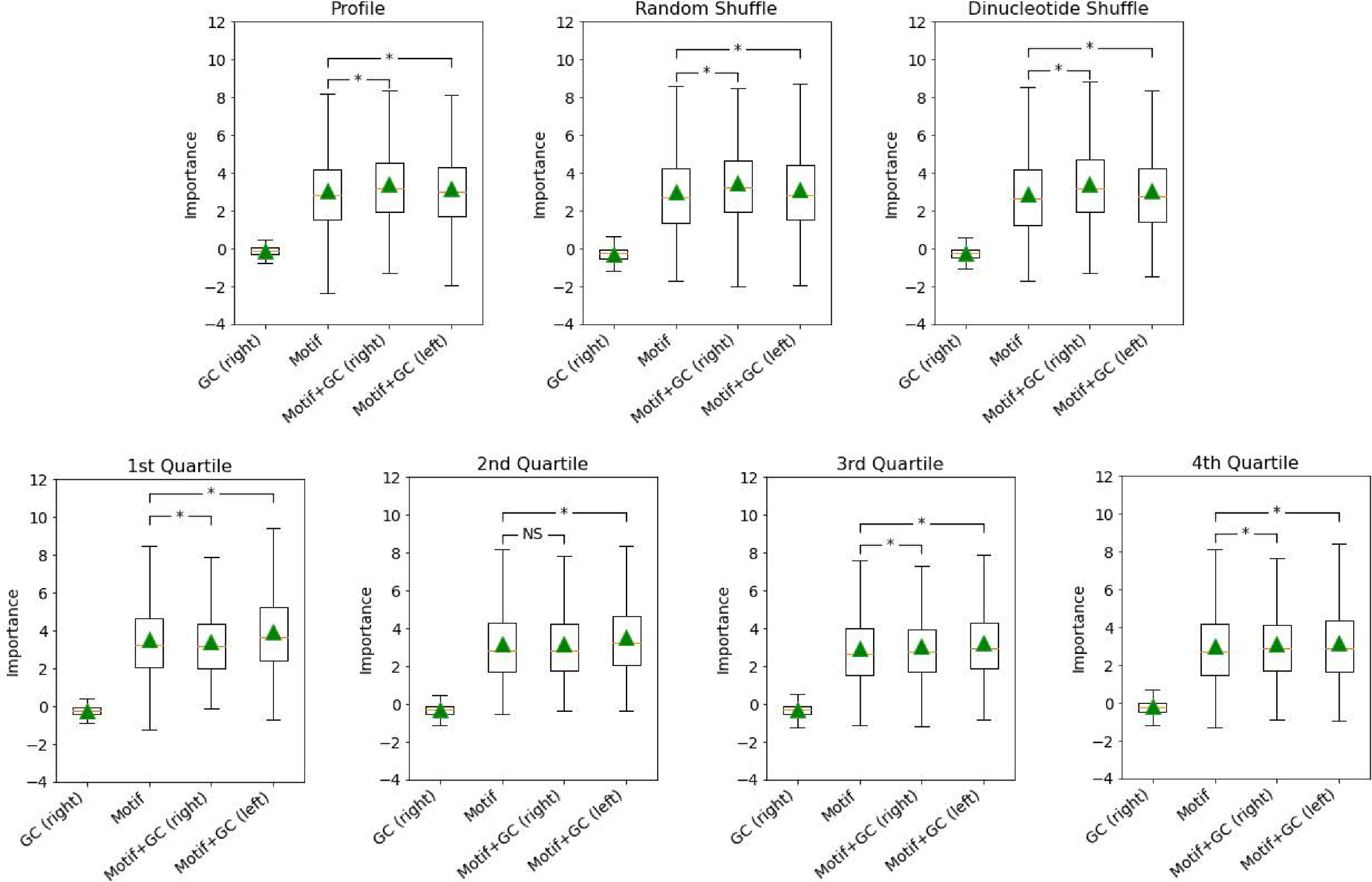

Supplement: S3 Fig — GIA was performed using different models of the approximate data distribution: profile, random shuffle, dinucleotide shuffle, and different binding score quartiles. Box plots show local importance for synthetic sequences with the top scoring 6-mer embedded in position 18-24 and GCGCGC embedded at positions 1-7 (Motif+GC, left) or positions 35-41 (Motif+GC, right). As a control, the GC content embedded at positions 35-41 without any motif is also shown. Green triangles represent the global importance. (TIF) [file pcbi.1008925.s004.tif]

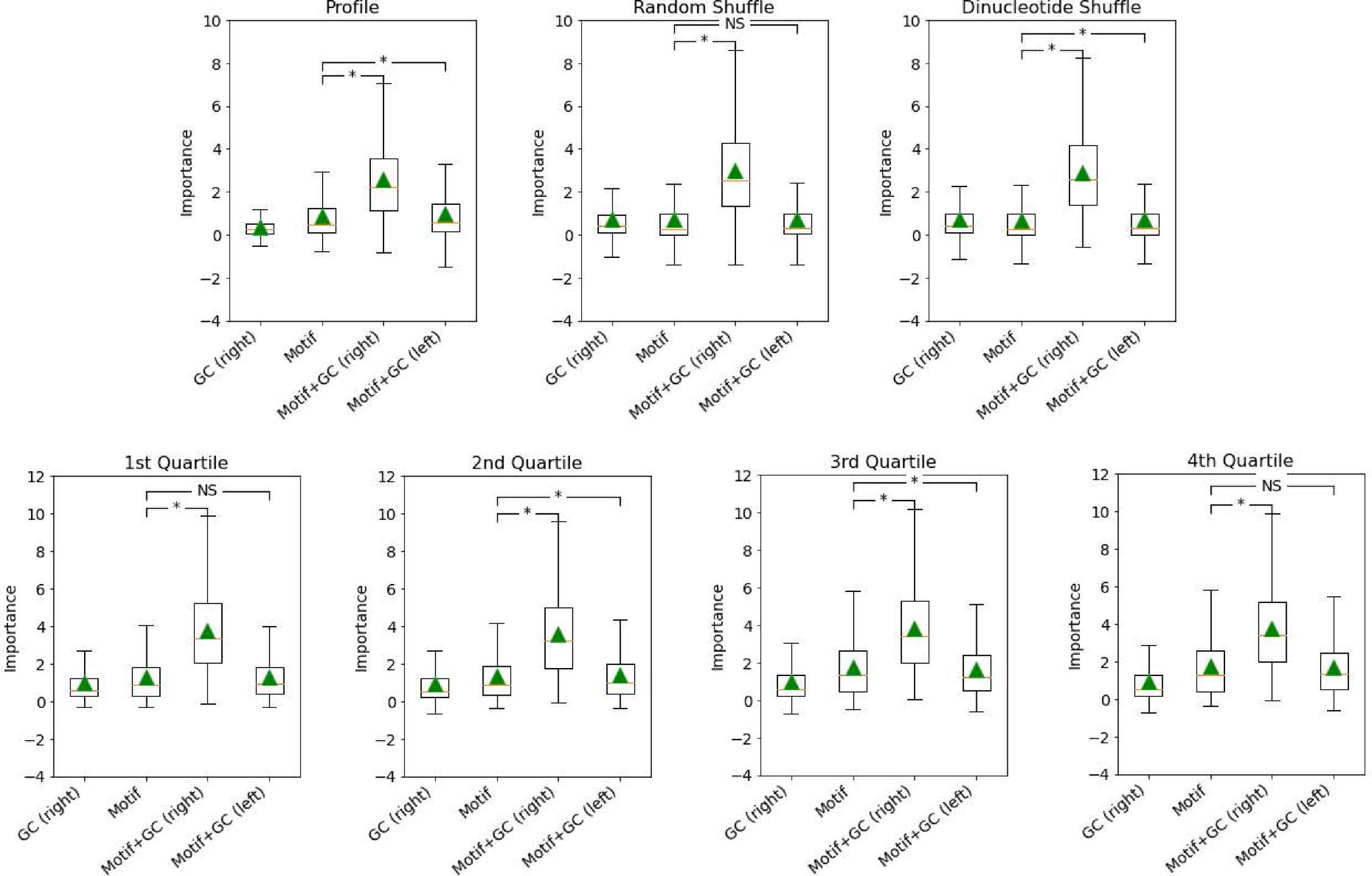

Supplement: S4 Fig — GIA was performed using different models of the approximate data distribution: profile, random shuffle, dinucleotide shuffle, and different binding score quartiles. Box plots show local importance for synthetic sequences with the top scoring 6-mer embedded in position 18-24 and GCGCGC embedded at positions 1-7 (Motif+GC, left) or positions 35-41 (Motif+GC, right). As a control, the GC content embedded at positions 35-41 without any motif is also shown. Green triangles represent the global importance. (TIF) [file pcbi.1008925.s005.tif]

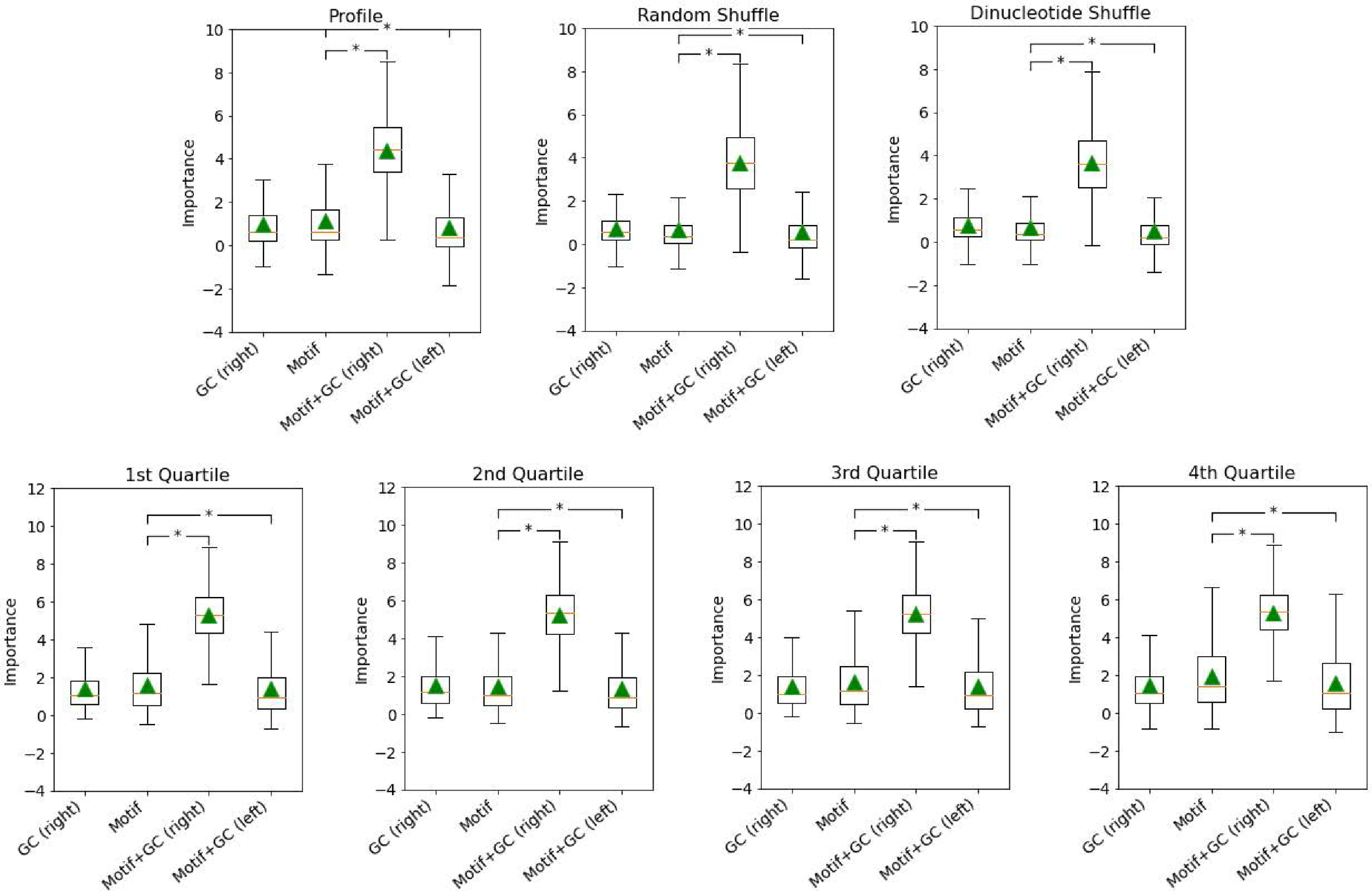

Supplement: S5 Fig — GIA was performed using different models of the approximate data distribution: profile, random shuffle, dinucleotide shuffle, and different binding score quartiles. Box plots show local importance for synthetic sequences with the top scoring 6-mer embedded in position 18-24 and GCGCGC embedded at positions 1-7 (Motif+GC, left) or positions 35-41 (Motif+GC, right). As a control, the GC content embedded at positions 35-41 without any motif is also shown. Green triangles represent the global importance. (TIF) [file pcbi.1008925.s006.tif]
