# Supplementary material for: Global importance analysis: An interpretability method to quantify importance of genomic features in deep neural networks
Source: PLoS Comput Biol. 2021 May 13;17(5):e1008925. doi: 10.1371/journal.pcbi.1008925 (PMC8118286; doi:10.1371/journal.pcbi.1008925)
Supplement: Attachment — Submitted filename: Response.pdf [file pcbi.1008925.s007.pdf]

We thank the reviewers for their insightful comments and suggestions. We address each concern below (reviewer comments are given in blue and our responses are in black) and in the revised manuscript (changes made in red text).

Response to Reviewer comments:

> Reviewer #1: Koo et al. present a novel algorithm to quantify the effect size, of putative patterns in genomic data, on model predictions (Global Importance Analysis). Further, they developed a new convolutional network to predict RNA-protein interactions (ResidualBind). I would recommend this work for publication with minor revisions.

We thank Reviewer1 for their support of this work.

> 1. You claim ResidualBind outperforms other methods on predicting RNA-protein interactions. Moreover, you mention predictions of other methods significantly increase when adjusting for secondary structures, while ResidualBind does not benefit. However, your description is not clear, if you compare ResidualBind to other methods while they are adjusted for secondary structures. I.e. e.g. ThermoNet might outperform ResidualBind in an adjusted setting.
>
> If this is the case and ResidualBind already includes the effects of secondary structures, it must miss effects the other methods account for. Could you theorise what these effects could be? If not, please mention (for the ones it applies), that they are already adjusted for secondary structures.

We agree with Reviewer 1 that this was not clearly described in the original manuscript. In the comparison of Figure 1, ResidualBind uses only sequence data, while the other models, such as ThermoNet, RCK, DLPRB, cDeepbind, use both secondary structures + sequence. In the revised manuscript, we have added a clarifying statement.

> 2. In case this provides additional information, could you report the Pearson's correlation for secondary structures (PU/PHIME), stratified by the probability "high vs low" to form those structures (e.g. median as a cut off or 1st vs 4th quartile)?

We ran this experiment as follows: 1. Calculated the ratio of paired/unpaired for each test sequence; 2. Sorted them in ascending order and split them into quartiles; and 3. We measured the performance (Pearson Correlation) within each bin. The results for the 4 bins are as follows:

	Quartile 1	Quartile 2	Quartile 3	Quartile 4
Mean	0.681	0.686	0.679	0.674
Std. dev.	0.183	0.182	0.185	0.187

We did not observe any statistically significant difference in performance for different ratios of paired/unpaired.

3. To further prove the performance boost of ResidualBind and its ability to recognise secondary structures without explicitly teaching them, you could show the performance difference of ResidualBind vs previous methods, using the 2009 set.
However, I leave it to the discretion of the authors.

Thank you for the suggestion. Indeed this would further prove the performance boost of Residualbind and its ability to recognize secondary structures. The main scope of the story is GIA and its various applications. We believe that the GIA hairpin experiment (Fig. 3) sufficiently demonstrates that ResidualBind is able to learn secondary structure preferences.

4. As I understand it, you compare ResidualBind with other methods while preprocessing for all with clip-transformation. While this might be outside of the scope of this work, did you test for the effect of log-transformation on the other methods?

We did not test other methods using the log-transformation. Comparison using CLIP-transformation was done because it is the current standard in the field -- all previous models were processed that way. Since ResidualBind's performance wasn't noticeably different, we opted to not perform a thorough comparison with previous methods using the log-transformation as it fell out of the central theme of the paper. Since the scope of the paper is to focus on GIA, we wanted to highlight GIA on sequences that exhibit interesting properties, which are usually in the high-binding score regime, and hence the need for the log-transformation.

5. Could you clarify if the 2009-RNAcompete Dataset was preprocessed the same way as the 2013-RNAcompete dataset?

Yes, the 2009-RNAcompete Dataset was preprocessed the same way as the 2013-RNAcompete dataset, using the log-transformation pipeline. In the revised manuscript, we have clarified this.

6. (Line 305) Add the table number. I assume you mean the supplementary table. Additionally, please provide the source for the CISBP-RNA database.

Done.

Reviewer #2: In this paper the authors presented (1) a residual CNN model (ResidualBind) for predicting RNA-protein binding from sequence and (2) a model interpretability approach (Global Importance Analysis) that evaluates the importance of a putative sequence feature by sampling group of sequences from a synthetic distribution and comparing the mean prediction scores of the sequences with and without the putative feature embedded over the population. The authors claimed that this approach enables quantitative hypotheses test and can be used for studying model learned global interactions among patterns and functions such as additive function or sequence context. Although the benchmarking result on RBP predictions for ResidualBind is promising, certain claims regarding the Global Importance Analysis (GIA) are not

convincing enough and only supported with ad-hoc synthetic data (which may lack generality) and limited comparison against existing interpretation methods along the same line.

We thank Reviewer2 for their thoughtful comments on our manuscript. In the revised manuscript, we have reframed GIA as a natural follow up to other interpretability methods to test hypotheses and performed additional experiments with other sequence sets to showcase GIA's robustness. See our response to each concerns below.

Major concerns:
1) Although the concept of being global and finding summarizing statistics over a population of sequences can be interesting, I'm concerned about the fully synthetic setup and the ad-hoc way the sequences are generated. It seems that the calculation of GIA is highly dependent on the selection of the embedding position i; however, no principled guidance on how to decide i is provided, neither do the rationalization of the choice of location 18-24. Given that the motif can appear in any locations in natural sequences, forcing them to be at a fixed position seems like an un-natural design choice, not to mention the case where there might be position specific patterns and an ad-hoc selected embedding position may easily break that pattern. On the other hand, modeling the contextual distribution is also nontrivial. Although the authors have noticed any uncaptured distribution modes or distribution mismatch could lead to misleading interpretation (especially when there is non-linear dependencies and interaction logic), they have not provided a principled and concrete guidance on how to deal with this problem. Listed options such as using PWM, dinucleotide shuffling can easily fall into the undesired scenario, and again no rationalization is provided why PWM is chosen for the analysis on ResidualBind. I would be more convinced if the authors at least try multiple design choices and empirically analyze their effect on the result.

GIA is highly dependent on the selection of the embedded position i by design. The position is selected based on a hypothesis. For instance, if one wanted to test whether there is a positional bias for the motif, then one can systematically perform GIA experiments where the motif is embedded in different positions. If one would like to marginalize out this nuisance parameter, then they could randomly embed the motif and average over all of the positions -- thus any positional bias would get averaged over. GIA provides a framework to test such hypotheses, but the hypothesis must come from the researcher. In the revised manuscript, we have reframed GIA as a method to test these hypotheses downstream of other interpretability methods, which often can help formulate hypotheses of what the network is learning.

In the original manuscript, we only demonstrated GIA using synthetic sequences sampled from a profile model. In the revised manuscript, we have added 6 additional null data distributions. These include a random shuffle of the observed sequences, a dinucleotide shuffle of the observed sequences, and randomly sampled subset of sequences from different quartiles of binding scores -- real sequences within the data distribution (but now embedded with a pattern-of-interest). We show in Figs. S1-S5 that GIA is robust to each of the explored sequence sets,

which include both synthetic and observed. This is primarily because it is getting the relative effect size based on a given sequence. We have also added multiple comments throughout the revised manuscript that describes intuition for the choice of a sequence model and potential choices in other settings, such as ChIP-seq.

Indeed, if there are non-linear dependencies and interaction logic, this can influence GIA's results. We have shown that GC-bias is one such confounder (Fig. 4) and RNA-secondary structure is for VTS1 (Fig. 3). GIA is not designed to help generate such hypotheses, but rather, once such a hypothesis is generated from attribution maps or other interpretability methods, then it could be tested downstream using GIA. In the revised manuscript, we have reframed GIA as an interpretability tool useful for analysis downstream of other interpretability methods, which can help to identify putative motifs or motif interactions.

> 2)The authors mentioned multiple times that GIA's global analysis over a population enables the study of feature interactions, however this claim is not well supported by the experiment. The example on counting the number of a repetitive motifs does not involve higher order interactions such as XOR logic or epistatic interaction, and the spacing example is too simple. Although ResidualBind is trained with real experimental data, these synthetic examples (repeating motifs and spacing) may never present in the train data so it is not fair to say that ResidualBind 'learnt' to do counting of motifs or spacing. Given that ResidualBind uses mean-pooling instead of max-pooling, it is not surprised to me that simply repeating a motif multiple times will result in higher activation, and one may observe similar correlation by looking at the activation instead of GIA score. More evidence is needed to show that this is something a model learnt and not an artifact of the CNN architecture, and that GIA is necessary for discovery of such pattern.

The GIA experiment for multiple motifs does not demonstrate feature interactions (as it is an additive model for motifs). This isn't a failure on the part of GIA to identify such interactions, but rather a consequence of what the model has learned. Indeed, the GIA experiment for the embedding of the VTS1 motif within the hairpin loop and the GC-bias experiments do indeed demonstrate such interactions (Fig. 3 and Fig. 4, respectively).

There are examples of multiple motifs present in sequences (identified via in silico mutagenesis). However, it is challenging to conclude how residualbind is integrating multiple binding sites from observing in silico mutagenesis maps on multiple sequences that have more than 1 binding site. GIA provides a way to perform interventional experiments to directly probe what residualbind has learned. Indeed, while there may not be an exact sequence with 3 RBFOX1 motifs embedded at the positions specified by Fig. 2, GIA experiments demonstrate the function mapped out by fitting the actual RNAcompete data. Prior to this, one would not know what kind of function the model has learned, on average. Of course this function is more complex and can be influenced by sequence context, but on average, it is additive.

For the question of learning the additivity of binding scores for multiple embedded motifs, the Reviewer suggested that ResidualBind and GIA may be relying simply on mean-pooling layer to

effectively count the number of motifs embedded, in which case the counting is much less to be credited to the architecture of ResidualBind. We agreed with Reviewer #2 that more evidence would be needed to support this claim, so we followed the suggestion and performed additional analysis with an amended architecture of ResidualBind where we replaced the mean-pooling layer by a max-pooling layer. We also did this for seven different sequence models. As we found (See Figure R1 below), the results remained very similar as in the original architecture (Figure S1), supporting the assumption that ResidualBind effectively learns the number of motifs in a more distributed way, as it does not depend on the presence of the mean-pooling layer.

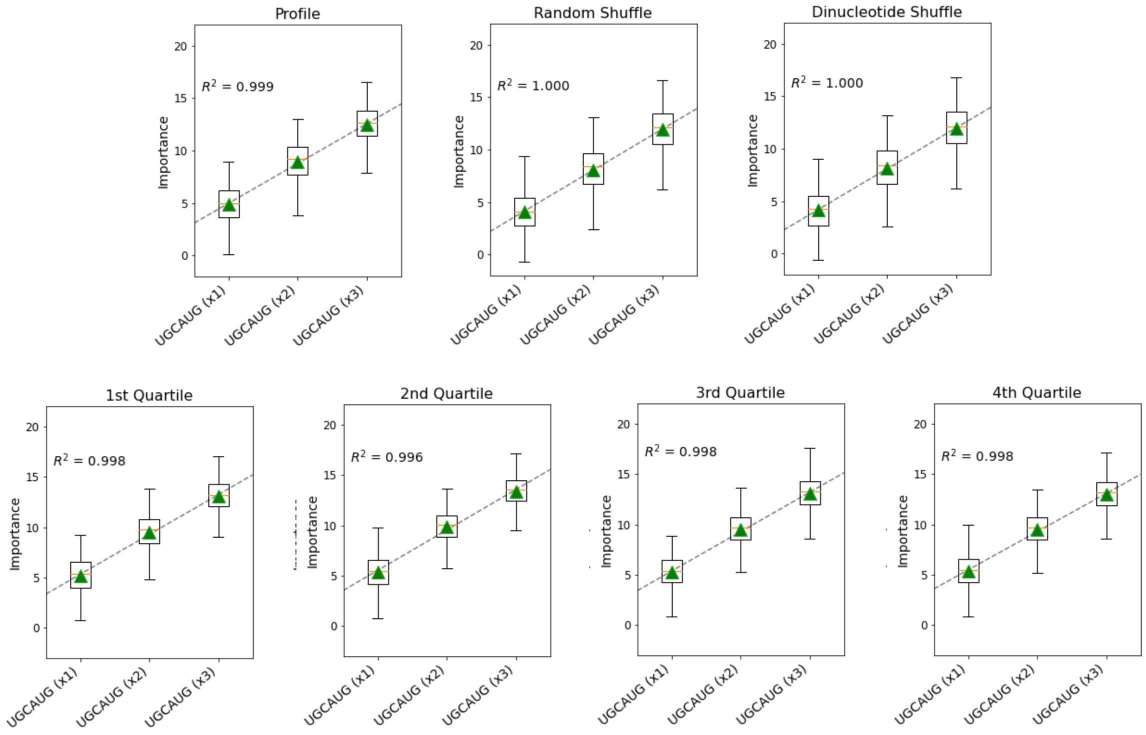

Figure R1 GIA experiment for additive binding sites of RBFOX1 using ResidualBind with max-pooling. Each boxplot demonstrates this using a different sequence set choice: profile model (used in the original manuscript and now Fig. 2), random shuffle of 1,000 randomly selected sequences in the test set, dinucleotide shuffle of 1,000 randomly selected sequences in the test set, and 1,000 observed sequences sampled randomly according to the quartile of the binding score. Notice the consistency in the GIA experiment across different sequence sets and that there remains a linear trend even with max-pooling in each case.

3)Although the methodology appears to be novel, each of its individual component has overlap with many existing studies, and the literature review and benchmark comparisons on prior methods is insufficient. For example the contrary to sequences without an embedded motif is very similar to integrated gradient [1] and DeepLIFT[2] which compare to a reference sequences. The use of multiple sequence samples to study distributional (instead of individual) feature importance has been introduced in

Max-Entropy [3]. Moreover, there exists multiple literatures that explicitly studies feature interactions in genomic neural networks with population level analysis, [4][5][6] and they have involved more comprehensive examples of interactions. The author stated that attribution-based methods cannot provide effect size of extended pattern which is not necessarily true, as the attribution scores of individual nucleotides can be summed up easily to make overall contribution score which has been shown effective in several literatures. It would be more convincing if the author can provide clarifications on distinctions between this work and existing literatures and rationales about why their design choices are superior to these existing methods, as well as quantitative comparison to the existing baselines other than in-silico mutagenesis.

[1] Axiomatic Attribution for Deep Networks, ICML, Sundararajan et. al, 2017
[2] DeepLIFT: Learning Important Features Through Propagating Activation Differences, ICML 2017
[3] Maximum entropy methods for extracting the learned features of deep neural networks, PLOS compbio, 2017
[4] Discovering epistatic feature interactions from neural network models of regulatory DNA sequences, bioinformatics, Greenside et al, 2018
[5] Visualizing complex feature interactions and feature sharing in genomic deep neural networks, BMC Bioinformatics, Liu et al, 2019
[6] Deep learning at base-resolution reveals motif syntax of the cis-regulatory code, bioarxiv, Avsec et. al, 2020

In the revised manuscript, we have added an extended section both in the introduction and discussion to highlight differences between different interpretability methods. Rather than being thought of as a competitor to these methods, we have reframed GIA as a downstream analysis to test hypotheses formulated by these other interpretability methods. The distinction really is that interpretability methods can often help to find features and feature interactions in a given sequence, albeit on an anecdotal basis. When an attribution map provides a noisy importance map with something that seemingly resembles a motif but there are other nucleotides containing some seemingly noisy importance (see Figure R2 below), it's not always clear cut what are the motifs? Are the other positions just spurious importance noise or sequence context? Are the singletons really important? One can observe multiple sequences, but they all tend to be just as noisy -- maybe a few are less noisy and some are more. GIA allows one to perform interventional experiments to quantify the effect size of each of these patterns in a controlled setting.

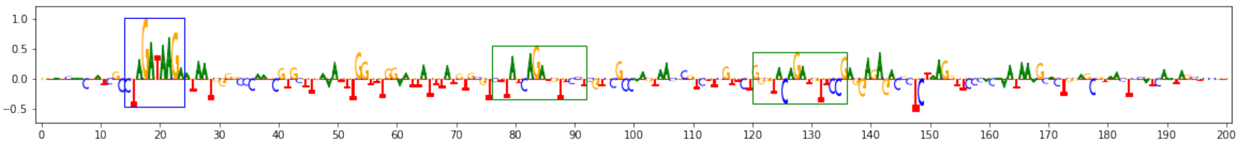

Figure R2. Deeplift importance scores for a sequence (taken from the deeplift github repository). Here a network was trained to classify synthetic data embedded with motifs for Tal1, GATA, and Tal1+GATA.

In terms of adding up attribution scores as a “motif” importance score, we have also added a comment in the discussion section to distinguish how GIA can provide more accurate effect sizes. There, we elaborate on how this assumes an additive model of nucleotides, for which binding sites may not necessarily follow due to nucleotide interactions within a motif and flanking nucleotides. There is a large literature of higher-order models that have been developed to explicitly model these interactions. DNNs can learn these via deeper layers -- as was indicated in learning secondary structure preferences directly from the sequence (Fig. 3) -- XOR interactions that cannot be learned by a convolutional layer alone. GIA quantifies the effect size of the whole motif pattern and thus makes no assumptions about the additive or non-additive nature of nucleotides, but rather uses the non-linear function learned by the neural network to quantify the whole motif importance. In the revised manuscript, we have added this argument in the discussion section.

> 4)It seems that the application of GIA requires prior knowledge about the location and the sequence which needs to be analyzed. Although it is helpful for validating putative features, it may not be very useful in general cases where the underlining mechanism and feature syntax are unknown. The author gave one example on an initio motif discovery; however, this is only applicable to single motif with known length and not easily generalizable to other complex features and interactions.

GIA is not useful when you don’t have a hypothesis. In our revised manuscript, we have reframed GIA as a tool to test hypotheses generated downstream of other interpretability methods. This should help resolve any confusion that GIA serves to replace other interpretability methods. GIA provides an avenue to test hypotheses, but it is limited in formulating the hypotheses.

> Minor concerns
> 1)The author referred to a bioarxiv literature which appears to be largely overlapping with the current submission and should be considered as the prior version of same submission. It would be better to remove such reference as it is confusing.

In the revised manuscript, we have removed the reference.

> 2)For the motif visualization of top GIA k-mer with in-silico mutagenesis, it is unclear if the L2-norm is taken w.r.t the GIA score or the change of GIA score after introducing mutation. It would make more sense if it’s the latter one, as an L2 norm of GIA score of all variants does not have information specific to the referenced wildtype and thus do not contain information about that nucleotide’s sensitivity. Also the scale of global importance values on fig 2e (-4,0) is difference than that of fig 2f (0,4).

The L2-norm is taken w.r.t. The change in GIA score after introducing mutation. In the revised manuscript, we have clarified this.

We apologize for the confusion. The scale of global importance values on fig 2e is negative because it is the change in global importance values w.r.t. Wild type. Fig. 2f provides the correct scale for the global importance values. In the revised manuscript, we have clarified this in the caption.

3)There are several typos/latex compilation errors in the text, e.g. line 222 p-value?0.01, and line305 missing table.

Fixed.
