# Supplementary material for: Global importance analysis: An interpretability method to quantify importance of genomic features in deep neural networks
Source: PLoS Comput Biol. 2021 May 13;17(5):e1008925. doi: 10.1371/journal.pcbi.1008925 (PMC8118286; doi:10.1371/journal.pcbi.1008925)
Supplement: Attachment — Submitted filename: Response.pdf [file pcbi.1008925.s008.pdf]

We thank the reviewers for their thoughtful comments and suggestions throughout the review process. We address each concern below (reviewer comments are given in blue and our responses are in black) and in the revised manuscript (changes made in red text).

> Reviewer #1: The authors addressed all my concerns. I recommend the manuscript for publication
>
> Reviewer #2: I appreciate that the authors have improved the manuscript based on prior reviews and most of the concerns are addressed.
>
> Specifically, the authors added clarifications in the discussion that GIA mainly serves as tool for downstream analysis of other interpretability methods. This is important as it helps the audience to identify which part of the practice in this paper is "artificially chosen" and needs customization in practice. Thus, it might be better if the authors could make this distinction clear even earlier in the paper (perhaps in introduction/method) whenever 'ad-hoc' design choices are used.

In the revised manuscript we have added a sentence in the Author Summary and the Introduction to specify that this is a follow up analysis to other interpretability methods. We have also added a more detailed discussion about the design choices in the paper and generalization to other data types in the discussion.

> It is good that the authors included 6 additional strategies for sampling synthetic sequences and showed more extensive results using max-pooling. I also appreciate the more comprehensive literature review on pre-existing methods of attribution-based and interaction interpretability tools, although the description of [39] is a bit misleading. As far as I know, DeepResolve uses multiple optimization samples and not just "one optimization run" to explore diverse patterns in the optimization landscape, and thus the limitation the author mentioned could be largely alleviated. It would be better if the authors consider modification of the corresponding paragraph for correctness.

We have replaced the misleading text with a more correct statement of DeepResolve in the introduction.
